# Platelet-activating factor induces cell cycle arrest and disrupts the DNA damage response in mast cells

**DOI:** 10.1038/cddis.2015.115

**Published:** 2015-05-07

**Authors:** N Puebla-Osorio, E Damiani, L Bover, S E Ullrich

**Affiliations:** 1 Department of Immunology, The University of Texas MD Anderson Cancer Center, Houston, TX, USA; 2 Dipartimento Scienze della Vita e dell'Ambiente, Universita Politecnica delle Marche, Ancona, Italy; 3 The University of Texas, Graduate School for Biomedical Sciences, Houston, TX, USA

## Abstract

Platelet-activating factor (PAF) is a potent phospholipid modulator of inflammation that has diverse physiological and pathological functions. Previously, we demonstrated that PAF has an essential role in ultraviolet (UV)-induced immunosuppression and reduces the repair of damaged DNA, suggesting that UV-induced PAF is contributing to skin cancer initiation by inducing immune suppression and also affecting a proper DNA damage response. The exact role of PAF in modulating cell proliferation, differentiation or transformation is unclear. Here, we investigated the mechanism(s) by which PAF affects the cell cycle and impairs early DNA damage response. PAF arrests proliferation in transformed and nontransformed human mast cells by reducing the expression of cyclin-B1 and promoting the expression of p21. PAF-treated cells show a dose-dependent cell cycle arrest mainly at G2–M, and a decrease in the DNA damage response elements MCPH1/BRIT-1 and ataxia telangiectasia and rad related (ATR). In addition, PAF disrupts the localization of p-ataxia telangiectasia mutated (p-ATM), and phosphorylated-ataxia telangiectasia and rad related (p-ATR) at the site of DNA damage. Whereas the potent effect on cell cycle arrest may imply a tumor suppressor activity for PAF, the impairment of proper DNA damage response might implicate PAF as a tumor promoter. The outcome of these diverse effects may be dependent on specific cues in the microenvironment.

Ultraviolet (UV)-mediated immunosuppression poses a major risk for skin cancer induction,^1, 2^ and many have reported that an essential mediator in this process is UV-induced platelet-activating factor (PAF; 1-alkyl-2-acetyl-*sn*-glycero-3-phosphocholine).^3, 4, 5^ PAF is a phospholipid, first discovered as a secreted component by activated innate immune cells,^6, 7^ that mediates its activity by binding to a G-protein-coupled receptor.^8^ It is involved in a variety of mechanisms including the release of histamine in activated leukocytes,^9, 10, 11^ anaphylaxis, and phagocytosis.^12^

Exposure to low doses of UV radiation activates PAF release by keratinocytes,^13, 14^ so it is likely that most of the population is regularly exposed to keratinocyte-derived PAF. In previous studies we showed that PAF upregulates both CXCR4 on mast cells and its ligand (CXCL12) on draining lymph node cells, promoting the migration of dermal mast cells from inflamed skin to the lymph nodes.^15^ Mast cells that reach the draining lymph nodes activate immune suppression by releasing interleukin 10.^16^ Blocking mast cell migration by using a CXCR4 antagonist, AMD3100, blocks UV-induced immune suppression and the induction of skin cancer.^15, 17^ No immune suppression is noted when PAF receptor-deficient mice (PAFR^-/-^) are exposed to UV radiation,^4, 5^ nor can one reconstitute immune suppression when PAFR^-/-^ mast cells are used to reconstitute mast cell-deficient mice.^18^ PAF also has a critical role in skin cancer induction and progression,^19, 20^ and this may reflect its capacity to both induce immune suppression and hamper DNA repair.^21^

Hanahan and Weinberg recognized the important roles inflammation and immune evasion play in the initiation of cancer.^22^ UV-induced PAF by activating immune suppression, retarding DNA repair and activating inflammation clearly constitutes an important hallmark for cancer induction. Supporting this idea is the observation that PAF is involved in a variety of other cancers besides skin cancer.^23, 24, 25, 26, 27^ Although we previously demonstrated that PAF suppresses the rate of DNA repair *in vivo*,^21^ little is known regarding the mechanisms involved. In this study we performed a series of experiments to determine how PAF affects DNA repair by examining important checkpoints that regulate DNA repair and cell cycle progression. We primarily used mast cells because of the critical role these cells have in UV-induced immune suppression and skin cancer induction,^15, 28^ and also because the dermis where they reside is targeted by UV-induced PAF.^18^

## Results

### cPAF impairs proliferation in mast cells

**Figure 1:**
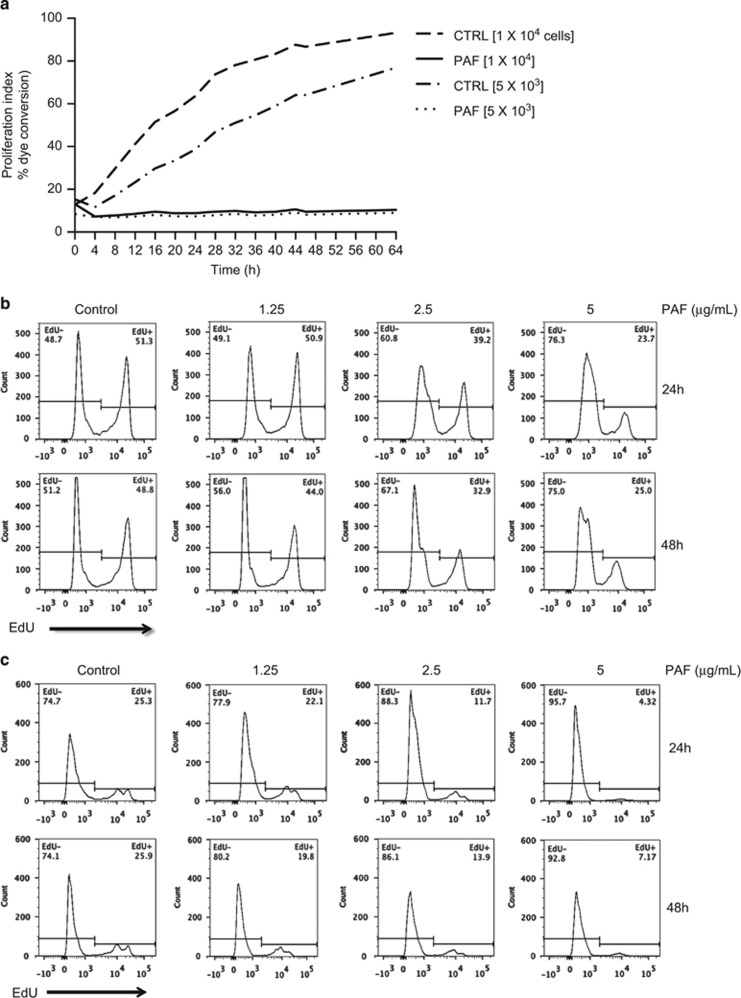
PAF suppresses cell proliferation. (**a**) HMC-1 cells, at the indicated densities per well, were treated with cPAF and proliferation measured by dye conversion. (**b**) Cells were treated with different concentrations of cPAF (0–5 *μ*g/ml) and proliferation was measured by EdU incorporation. Cells were harvested 24- and 48-h post-cPAF treatment. (**c**) Effect of cPAF on the proliferation of normal mast cells was measured by EdU incorporation

Conflicting studies show that PAF activates or inhibits cell proliferation, suggesting potential roles in tumor promotion or tumor suppression.^29^ To understand the definitive role of PAF on transformed human mast cells (HMC-1), we cultured HMC-1 cells with 5 *μ*g/ml of carbamyl PAF (cPAF), a non-hydrolysable bioactive PAF agonist, and observed a significant decline in cell proliferation (Figure 1a). Similarly, the rate of incorporation of the thymidine analog ethynyl deoxyuridine (EdU) into DNA declined after cPAF exposure, in a dose- and time-dependent manner (Figure 1b). PAF treatment also had a similar effect in nontransformed cells. Normal mast cells were isolated from a buffy coat and treated with cPAF as described above. Although these cells had a lower basal rate of cellular growth, cPAF treatment also induced a dose-dependent decrease in proliferation (Figure 1c). These results indicate that the cellular response to cPAF is not affected by transformation.

### cPAF induces cell cycle arrest at G2–M

**Figure 2:**
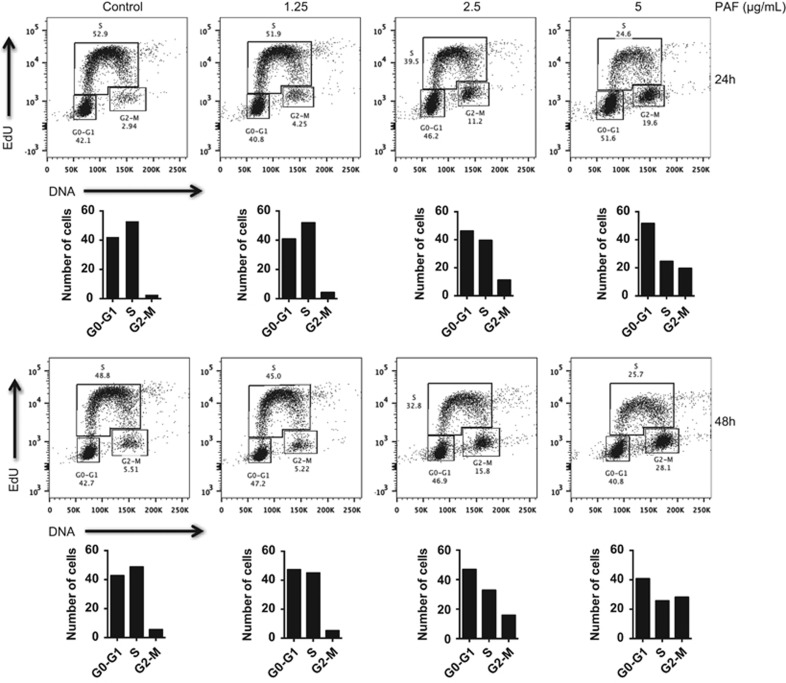
cPAF disrupts cell cycle progression. HMC-1 cells were treated with different doses of cPAF, and incubated for 24 and 48 h. Cells positive for propidium iodide but negative for EdU were increased at G2–M and G0–G1. The number of double positive cells (PI^+^ and EdU^+^), indicative of DNA synthesis, was decreased in cells treated with cPAF

To identify the compartments of the cell cycle affected by cPAF, we stained EdU^+^ cells with propidium iodide and calculated the percentage of proliferating cells. We detected a dose-dependent reduction in DNA synthesis (S phase), and arrest at G2–M, 24 and 48 h after cPAF treatment (Figure 2). A less pronounced but similar effect was observed at the G0–G1 phase 24 and 48 h after cPAF treatment. This indicates that cPAF causes a long lasting effect at G2–M, reminiscent of chemotherapeutic drugs affecting G2–M transition.^30, 31^

**Figure 3:**
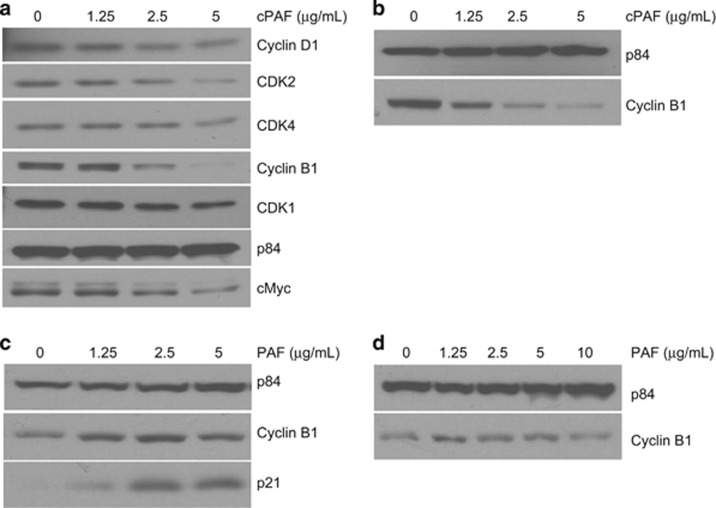
cPAF decreases protein levels of key components of the cells cycle. (**a**) Protein extracts from cells exposed to different concentrations of cPAF for 24 h were analyzed by immunoblotting. A noticeable decrease as a function of concentration response is observed in cyclin-B1, CDK2/4 and c-Myc. (**b**) cPAF also induced a decrease in cyclin-B1 expression in normal, nontransformed mast cells, similar to that observed in transformed mast cells. (**c**) Similar to cPAF, natural occurring PAF also induced an increased expression of p21 within 3 h after exposure; however, the effect on cyclin-B1 was more evident at 6-h post exposure and higher concentration of natural PAF. (**d**) p84 expression was used as the loading control

Because cPAF largely arrests cells at G2–M, we sought to determine the targets altered during this process. We used fluorescently-labeled cell sorting to separate EdU^-^ from EdU^+^ HMC-1 cells 24 h after cPAF exposure. Protein lysates were analyzed using reverse phase protein arrays (RPPA).^32^ The RPPA results (Supplementary Figure 1) indicated that cPAF reduces the expression of CDK1 (cyclin-dependent kinase) and cyclin-B1, critical for G2–M transition, and of cyclins D1 and E1, E2F1, and CDK2, essential at G0–G1. These results agree with the fluorescence-activated cell sorting (FACS) analysis indicating that cPAF arrests cells mainly at G2–M and G0–G1 (Figure 2). These observations were confirmed by immunoblotting, in which a dose-dependent decline was observed in cyclin-B1 and CDK1 expression. We also observed a slight decrease in cyclin-D1 and CDK2/4, which are essential at G0–G1. In addition, the highest dose of cPAF induced a consistent decrease in c-Myc (Figure 3a), likely affecting the expression of its downstream target, cyclin-D1.^33^ Similar changes were observed for cyclin-B1 after exposing primary mast cells to cPAF (Figure 3b), thus confirming that the effects of cPAF were not restricted nor affected by transformation. Next, we conducted experiments to confirm that the effect of cPAF was similar to that of naturally occurring PAF. Natural PAF has a shorter life and is more susceptible to enzymatic degradation as compared with cPAF;^34^ this makes cPAF a reliable choice to study PAF effector functions. As expected, natural PAF showed a strong activation of p21 3-h post exposure, and a decrease of cyclin-B1 6-h after exposure (Figures 3c and d). These results indicate that the effector function of cPAF closely resembles that of natural PAF, although the latter is more susceptible to degradation and thus its effects occurred within shorter incubation times.

### cPAF effect on the G2–M mitotic complex

**Figure 4:**
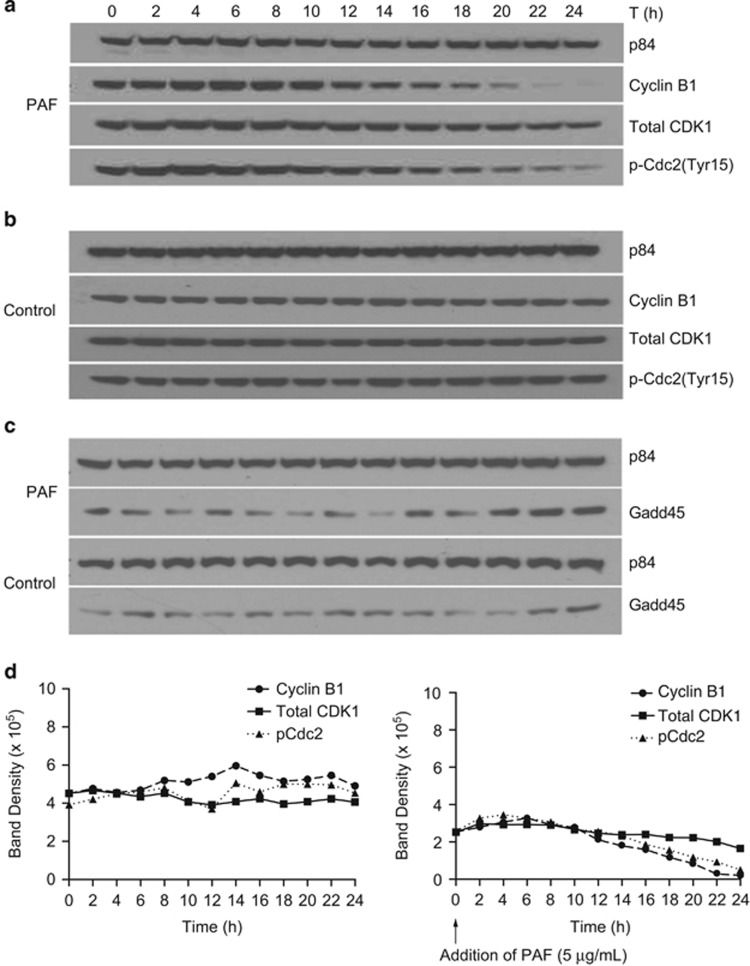
cPAF disrupts the cyclin-B1/CDK1 mitotic complex. (**a**) Cells were collected every 2-h post-cPAF treatment and expression of cyclin-B1, total CDK1 and phosphor-Cdc2(tyr15) determined by immunoblotting. (**b**) Nontreated control samples harvested at the same time points. (**c**) Expression of GADD45 in cPAF-treated and control cells. p84 expression was used as the loading control. (**d**) Blot density for cyclin-B1, total CDK, and phospho Cdc2(Tyr15), was determined using ImageJ on blots from control and cPAF-treated cells

The G2–M DNA damage checkpoint prevents eukaryotic cells from entering mitosis allowing for proper repair of damaged DNA, thereby preventing genomic instability.^35^ The G2–M mitotic transition is regulated by cyclin-dependent kinase 1 (CDK1) that associates with the regulator cyclin-B1. The phosphorylation of Cdc2 at Tyr15 measures the activation status of Cdc2 during progression into mitosis.^36^ Since a significant decrease in cyclin-B1 and CDK1 was observed 24 h post-cPAF treatment (Figure 3), we wanted to understand how the expression of these proteins was modulated during the first 24 h of cPAF exposure. We found a steady decline in cyclin-B1, total CDK1 and p-Cdc2 (Tyr15) expression starting at 10-h post-cPAF treatment (Figure 4a). The levels of cyclin-B1 and p-Cdc2 (Tyr15) reached their lowest at 22–24-h post-cPAF treatment, as opposed to the control samples (Figure 4b). The cPAF-induced reduction in these proteins was similar in HMC-1 cells synchronized at G0 by serum starvation (Supplementary Figure 2). Because serum deprivation affected the viability of HMC-1 cells, but not the cPAF-induced modulation of cyclin-B1, CDK1, and p-Cdc2 (Tyr15) expression, we decided to avoid it in our subsequent experiments. To quantify differences in protein expression, we obtained normalized blot densities for cyclin-B1, CDK1, and p-Cdc2 (Tyr15; Figure 4d). Our results show that after adding cPAF, cyclin-B1 expression peaked at 6 h and gradually decreased over the remaining time, and total CDK1 peaked at 2 h and followed a nearly flat trajectory during the 24-h cPAF exposure. CDK1 expression did not surpass the peak of p-Cdc2 (Tyr15), an indication of CDK1 inactivity, at earlier time points. However, the expression of CDK1 remained higher than p-Cdc2 (Tyr15) 14-h post-cPAF exposure. These results indicate that while the phosphorylation of Cdc2 decreases, an indication of active CDK1, the level of cyclin-B1 does not recover to resume cell cycle progression. This appears to be sufficient to keep cells arrested at G2. To assess whether an inhibitor of the CDK1/cyclin-B1 complex resulted from cPAF exposure, we analyzed the expression of growth arrest and DNA damage-inducible 45 (GADD45) during the same 24-h period. Our results indicate an increased expression of GADD45 at 22–24 h, which coincides with the decreased expression of CDK1/cyclin-B1, suggesting that GADD45 might be affecting this complex (Figure 4c). Finally, to determine whether cPAF activates the MAPK pathway in HMC-1 cells, as has been shown with other innate immune cells,^37^ we measured the activation of extracellular signal-regulated kinase (ERK). Our results indicate a decrease in activated ERK starting 8-h post-cPAF exposure (Supplementary Figure 3), suggesting that cPAF might also affect cell cycle entry through this mechanism.^38^

### cPAF exposure increases p21 expression

**Figure 5:**
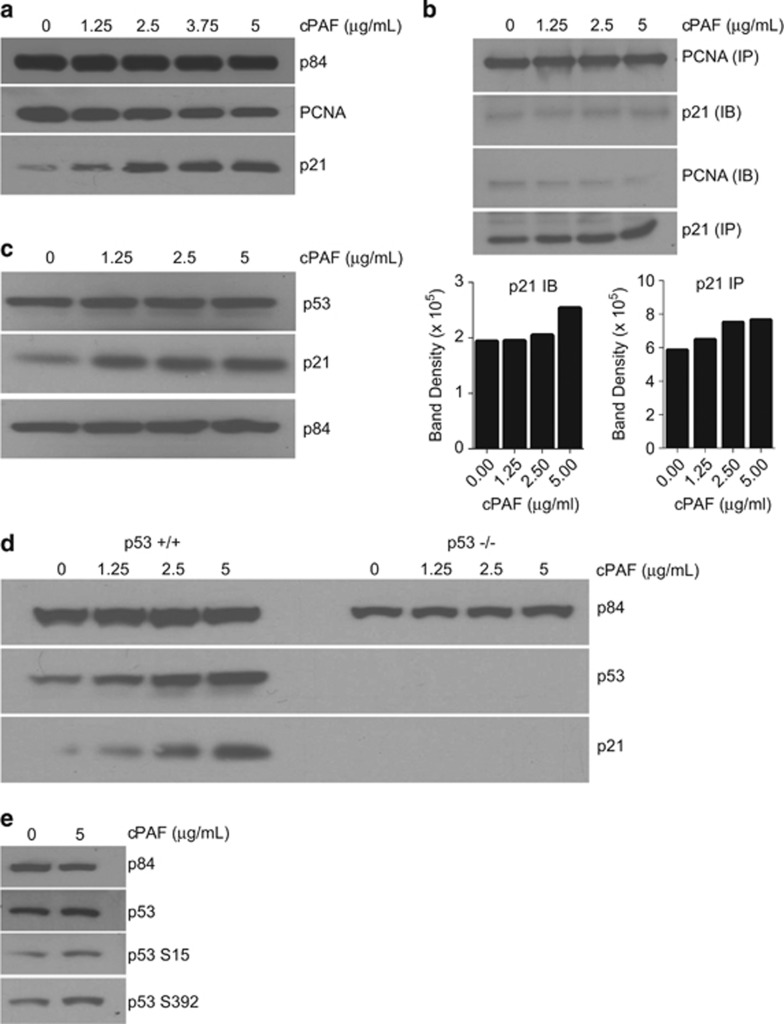
cPAF increases the expression of p21 in a p53-dependent manner, and affects its interaction with PCNA. (**a**) Expression of PCNA and p21 in cells treated with different doses of cPAF and harvested 24-h post treatment. (**b**) Lysates from cPAF-treated cells were immunoprecipitated with anti-PCNA (PCNA IP) and immunoblotted wi th anti-p21 (p21 IB), or immunoprecipitated with p21 (p21 IP) and blotted with anti-PCNA (PCNA IB). Histograms represent normalized band density as determined by ImageJ analysis of the Western blots. (**c**) cPAF induced an increased expression of p21 with no visible changes in p53 expression in mast cells; however, the effect of cPAF is more evident in HCT-16 cells carrying wild-type p53, which show a robust expression of both p21 and p53 (**d**, left panel); Saos-2 cells were used as p53-null control (**d**, right panel). (**e**) cPAF also induced the expression of phosphorylated p53 at S392, and a subtle increased in p53 S15, as compared with the control. p84 expression is used as the loading control

Because p21 regulates cell proliferation through its involvement with proliferating cell nuclear antigen (PCNA), we determined whether cPAF affected this interaction. cPAF induced a measurable reduction in PCNA expression (Figure 5a). Conversely, the expression of p21 showed a dose-dependent increase. These findings indicate that cPAF may reduce cell proliferation by unbalancing the interaction between PCNA and p21 to the extent that abundance of p21 may result in higher occupation of PCNA sites, thereby preventing PCNA-driven cell proliferation.^39^ To determine whether this was the case, we performed immunoprecipitation assays, capturing with PCNA antibody and blotting with anti-p21 (Figure 5b). As indicated by immunoblotting and densitometry analysis, we found that cPAF induced a dose-dependent increase in p21 bound to PCNA (Figure 5b). Similarly, when anti-p21 was used as the capture antibody and anti-PCNA antibody for immunoblotting, we observed higher PCNA levels in control samples as opposed to cPAF-treated samples (Figure 5b). These findings indicate that cPAF-induced p21 expression likely leads to the occupation of PCNA sites, impairing cell proliferation.

Next, we determined if cPAF-induced expression of p21 was associated with p53 activation. Although we observed a discrete increase in p53 expression with robust expression of p21 in PAF-treated mast cells (Figure 5c), we wanted to determine whether PAF-induced p21 expression was associated with p53 upregulation in different cell types. We exposed HCT-16 cells, carrying wild-type p53, and p53-deficient Saos-2 cells to different doses of cPAF. Our results indicate that while cPAF failed to induce p21 expression in Saos-2 cells, it induced a robust expression of p53 and p21 in HCT-16 cells. These results indicate that cPAF-induced expression of p21 is p53 dependent (Figure 5d). Next, to identify the effect of cPAF on the phosphorylation of p53, we use a panel of antibodies against the following phosphorylation sites: S15, S9, S37, S6, S46, S20, S392, and T81. We observed that cPAF increased the expression of phosphorylated p53 at S392 (Figure 5e), probably enhancing its binding capacity to the promoter region of p21 as described by Kapoor and Lozano.^40^ Taken together, these findings unravel a novel and yet undiscovered role of cPAF on key proteins critical for cell proliferation that indicate a potential role in tumor suppressor mechanisms.

### cPAF drives transformed mast cells into apoptosis

**Figure 6:**
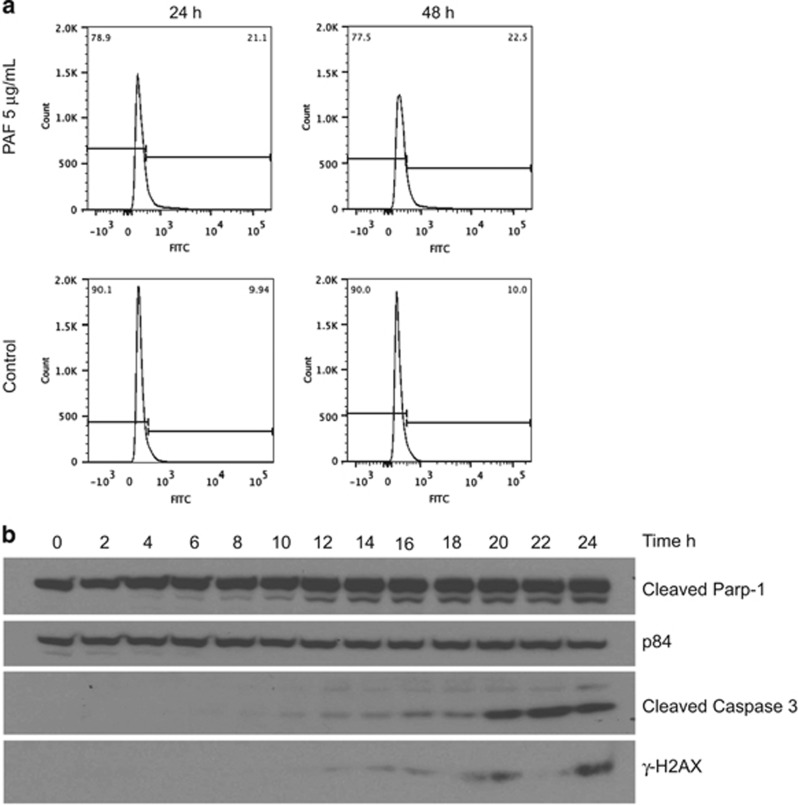
cPAF induces expression of pro-apoptotic markers. (**a**) HMC-1 cells were treated with 5 *μ*g/ml of cPAF and the number of apoptotic cells was measured 24- and 48-h post-cPAF treatment. (**b**) HMC-1 cells were treated with 5 *μ*g/ml of cPAF and samples were harvested at the indicated time points. Expression of cleaved Parp-1, cleaved Caspase and *γ*-H2AX was determined by Western analysis. p84 expression is used as the loading control

Because cPAF treatment impairs mast cell proliferation, it was important to investigate the fate of these cells. Using FACS and TUNEL (terminal deoxynucleotidyltransferase-mediated dUTP-biotin nick end labeling) analyses, we observed that cPAF-exposed HMC-1 cells undergo apoptosis in a concentration-dependent manner (Figure 6a). We also measured the expression of cleaved PARP-1 and cleaved caspase 3 by immunoblotting following cPAF exposure and observed a steady induction of cleaved PARP-1 starting at 6 h and a gradual increase in cleaved caspase 3 starting at 10 h (Figure 6b). We also determined the accumulation of fragmented DNA from apoptotic cells by measuring the expression of *γ*-H2AX during the same time period. Our results show that, in line with the expression of cleaved PARP-1 and caspase 3, the dying cells also accumulate increasing amounts of phosphorylated H2AX (Figure 6b). This indicates that cPAF-induced impaired cell proliferation is followed by apoptosis.

### cPAF impairs the DNA damage response

**Figure 7:**
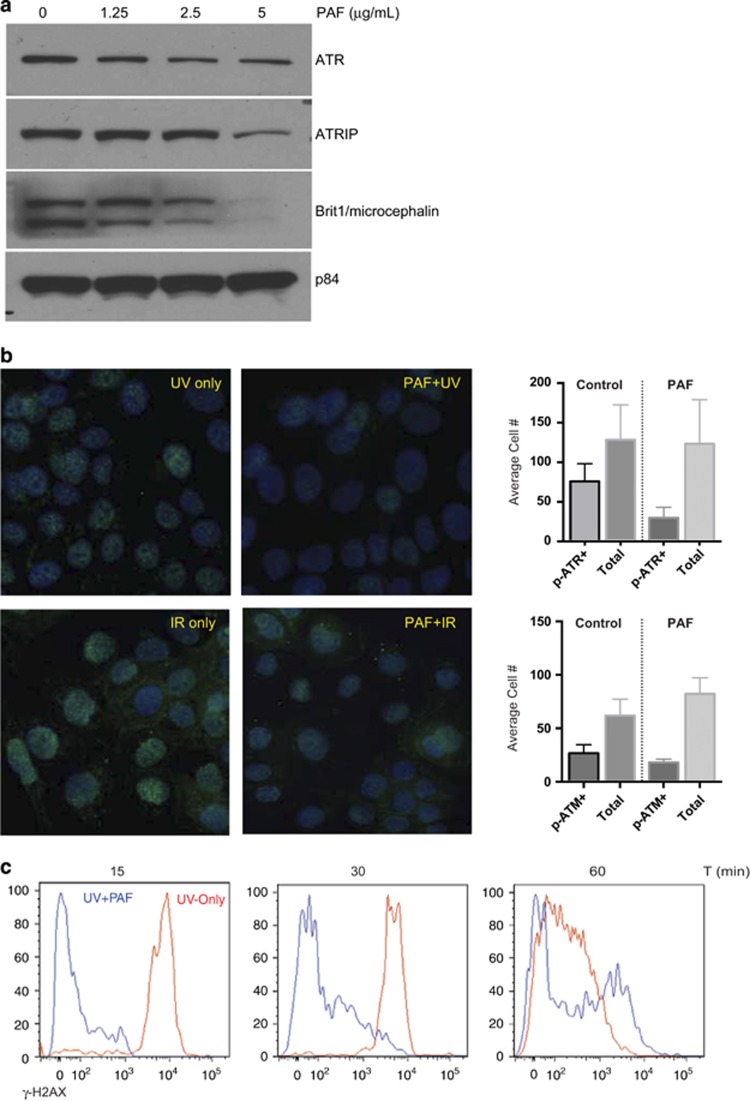
cPAF disrupts the expression of key components of the DNA repair mechanism. (**a**) HMC-1 cells were treated with different doses of cPAF and harvested 24-h post treatment. Expression of ATR, ATR-interacting protein, and Brit1 was determined by Western analysis. (**b**) HaCat cells were exposed to UV or IR, or pre-treated with 5 *μ*g/ml of cPAF, and then exposed to UV or IR. Immunofluorescence using antibodies against phosphorylated forms of ATR (S428) and ATM (S1981) was used to visualize the number of cells with positive foci. (**c**) Histograms represent the average number of p-ATR+ and p-ATM+ cells in the cPAF-treated or control cultures. (**d**) HMC-1 cells were exposed to UV radiation (red) or pre-treated with 5 *μ*g/ml of cPAF and then exposed to 200 J/m^2^ UV radiation (blue). The expression of *γ*-H2AX 15, 30, and 60 min after UV exposure was measured by flow cytometry

The process of DNA repair is inherently linked to cell cycle progression and its checkpoints. To determine whether cPAF also affected components of the DNA damage repair mechanism, we measured the protein expression of several factors critical in this process, including ATR, ATR-interacting protein and microcephalin/BRIT-1. Our results show that cPAF decreases BRIT-1 levels, critical for the repair of ionizing radiation and UV-related DNA damage, in a concentration-dependent manner (Figure 7a). Similarly, cPAF induced a moderate decrease in ATR and ATR-interacting protein in HMC-1 cells. This indicates that cPAF might impair a proper DNA damage response if cells are exposed to damaging agents. To test this, we asked whether cPAF affected the localization of p-ATR and p-ATM to sites of DNA damage. Briefly, we placed keratinocyte monolayers (HaCat) onto multi-chamber slides; the cells were pre-incubated with cPAF for 24 and 48 h followed by UV or ionizing radiation (IR). Immunofluorescence staining demonstrated that cells exposed to cPAF followed by UV exposure, had a lower number of p-ATR (S428)-positive foci, as compared with the controls (Figures 7b and c). Similarly, cells that were exposed to IR had a lower number of localized p-ATM (S1981)-positive foci when compared with the controls (Figures 7b and c). These observations indicate that cPAF disrupts both the cell cycle and the DNA repair mechanism, potentially increasing the risk of genomic instability.

The recruitment of the phosphorylated form of histone H2AX (*γ*-H2AX) is another indication of a rapid DNA damage response mechanism after cells are exposed to genomic insults. Hence, the expression of *γ*-H2AX in cPAF- and UV-exposed HMC-1 cells was analyzed. Our results show that cPAF treatment delays the expression of *γ*-H2AX in UV-treated mast cells, compared with cells exposed only to UV radiation (Figure 7d). This suggests that the presence of cPAF hampers the repair of DNA damage induced by UV exposure.

## Discussion

Exposure to moderate UV doses results in the release of PAF by irradiated keratinocytes.^13, 41^ Previous studies have shown that PAF has an important role in UV-induced immune suppression^3, 4, 5, 42^ and skin carcinogenesis, in part by suppressing DNA repair.^21^ Here we demonstrate that PAF profoundly affects key components that regulate the cell cycle and DNA damage response in mast cells and keratinocytes. Contradicting previous reports indicating that PAF promotes proliferation in keratinocytes^43^ and metastasis in a variety of tumor cells,^29^ we demonstrate that in HMC-1 cPAF, a non-hydrolysable PAF analog, suppresses rather than accelerates cell growth (Figure 1a), suggesting a potential function in tumor suppression. cPAF also affected normal mast cells demonstrating that its effect was not exclusive to transformed cells (Figures 1b and c).

FACS analysis showed that cPAF exposure induces a potent reduction in DNA synthesis, and a significant arrest at G2–M. However, cPAF only had a discrete effect at G0–G1 (Figure 2). Our initial findings obtained by RPPA identified key regulators of the cell cycle affected by cPAF (Supplementary Figure 1). RPPA analysis showed that cPAF downregulated proteins actively involved in the cell cycle including cyclin-B1, CDK1, cyclins D1/E, and CDK2. We next observed that cPAF promotes a continuous degradation of cyclin-B1 in a concentration-dependent manner to almost null levels 24- and 48-h post exposure (Figure 3). These results and our previous observations indicated that the cPAF-induced cytostatic effect in HMC-1 cells is predominantly at G2–M through the disruption of the CDK1/cyclin-B1 mitosis-promoting complex,^44, 45, 46, 47^ suggesting that cPAF-induced reduction of cyclin-B1 expression forces cells to exit from mitosis.^46^ The effect of cPAF on cyclin-B1 in transformed cells underscores potential therapeutic applications of this phospholipid, since cyclin-B1 is a regulator of the CDK1/cyclin-B1 complex, and is critical for mitotic progression.^45, 46^ In addition, cPAF also induced a consistent decrease in c-Myc expression throughout all our experiments, providing further evidence it is a potent suppressor of cellular proliferation.

In our experiments the effects of cPAF resemble those of naturally occurring PAF, with few minor differences. Whereas most of cPAF-induced changes occurred at the end of 24 h of exposure, natural PAF induced changes in p21 and cyclin-B1 expression within the first 6 h of exposure. This difference can be explained by the incorporation of the carbamyl radical to PAF, which makes it more resistant to metabolic degradation without compromising its biological activities.^34^

To understand how cPAF affected key components regulating the G2–M mitotic complex, we studied the fluctuation of cyclin-B1, CDK1, and p-Cdc2 (Tyr15) during a 24-h time frame. Our results clearly indicate that cPAF depresses expression of cyclin-B1, coinciding with the reduced phosphorylation of Cdc2 at Tyr15 and reduced expression of total CDK1 starting 10 h following cPAF addition. As observed by the peak in the expression of cyclin-B1, and compared with the controls, cPAF seems to accelerate the cells into mitosis; indeed, cyclin-B1 reaches its maximum after 6 h but never recovers to resume cell cycle. The effect of cPAF on cyclin-B1 is unique because the decrease in p-Cdc2 (Tyr15) at 22–24-h post-cPAF exposure shows that CDK1 is readily active to resume cell cycle progression, however, the decreased expression in cyclin-B1 likely impairs this process and blocks the cells from entering mitosis. Another mechanism that prevents cell cycle entry is the reduction in ERK activity,^38^ and in this study we found that cPAF exposure reduces the expression of activated ERK (Supplementary Figure 3). This finding is in contradiction to previous observations in colorectal cell lines showing PAF-induced increased activation of ERK and p38MAPK. This difference is probably due to the diverse effects of PAF in different cellular contexts.^48^

Our studies provide further evidence that the CDK1/cyclin-B1 complex could also be affected by the dual induction of p21 and GADD45 after cPAF exposure. First, PAF-induced p53-dependent induction of p21 leads to the disruption of PCNA-controlled cell proliferation, contributing to arrest at G0–G1. Second, cPAF-induced increased expression of GADD45, which is described as an inhibitor of the G2–M complex,^49, 50, 51^ and is involved in its checkpoint after UV damage,^52^ could be having a major role in the cell cycle arrest at G2.^53^ The evidence we show here indicates that cPAF might be affecting other compartments of the cell cycle, however our results show that by disrupting cyclin-B1, G2–M is central to cPAF activity in HMC-1 cells. On the other hand, the decreased cell proliferation and the effect at G2–M also coincide with the cPAF-induced reduction in c-Myc, cyclin-D1, and the cyclin-dependent kinases CDK4 and CDK2 (Figure 3a).

As a consequence of the cPAF-induced arrest at G2–M, the HMC-1 cells show a robust expression of cleaved PARP-1 combined with an increase in cleaved caspase 3 and *γ*-H2AX (Figure 6). This indicates that PAF-treated cells stall at G2–M, undergo mitotic catastrophe,^54^ and accumulate damaged DNA originated from apoptotic fragmentation^55^ or stalled DNA replication.^56^ Thus, cPAF-induced cell cycle arrest at G2–M is followed by apoptosis in HMC-1 cells.

An effective DNA damage response mechanism is critical to maintain genomic stability. Our previous studies suggest that cPAF might be affecting the capacity of UV-exposed cells to efficiently repair damaged DNA.^21^ Here we show that cPAF exposure induces a significant reduction in critical DNA repair components including ATR, ATR-interacting protein, and Brit1. Using this as reference we tested whether cPAF affected the capacity of cells to mount an early DNA damage response. HMC-1 cells incubated with cPAF and exposed to either UV or ionizing radiation, showed an important decrease in the recruitment of DNA damage response proteins p-ATR (S428)^57, 58^ and p-ATM (S1981),^59^ respectively. Therefore, although extended exposure to cPAF leads to mitotic catastrophe and apoptosis, early events indicate that cPAF disrupts a proper DNA damage response upon genomic insult. This suggests that while PAF is released during chronic inflammatory conditions, or during direct exposure to UV-damaging agents (i.e., sunlight) it is likely that neighboring cells are affected in their capacity to reduce the deleterious effects of these agents.

PAF is released by keratinocytes following low to moderate UV exposure,^13, 14^ suggesting that keratinocytes release PAF on a regular basis. Most of the studies examining the effect of PAF on immune function and carcinogenesis suggest it has a negative effect, so the evolutionary advantage of daily release of this inflammatory mediator that has such deleterious effects is not readily apparent. The findings presented here may shed some light on the role of PAF in normal and inflamed skin. UV radiation is highly mutagenic and after an acute exposure to low to moderate doses of sunlight, PAF, by inducing cell cycle arrest and promoting apoptosis, may help accelerate the removal of DNA damaged cells from the skin, thus promoting genomic stability. On the other hand, after chronic UV exposure, and coupled with UV-induced inactivation of p53,^60^ PAF suppresses effective DNA repair and promotes tumor growth. By depressing DNA repair, PAF may also augment immune suppression, as studies by Kripke *et al.*^61^ clearly demonstrated that DNA lesions, particularly pyrimidine dimer formation activate immune suppression. We suggest that PAF has dual functions. In normal skin, it can promote apoptosis and maintain normal homeostasis. However, in chronically irradiated inflamed skin, where one finds UV-induced inactivation of normal tumor suppressor pathways (i.e., p53, PTEN), PAF acts as a classic mediator of inflammation, and through its ability to depress DNA repair contributes to skin cancer induction.

## Materials and Methods

### Cell culture

HMC-1 cells (kindly provided by Dr. JH Butterfield, Mayo Clinic, Rochester, MN, USA)^62^ were cultured using RPMI-1640 medium enriched with vitamins, non-essential amino acids, and fetal bovine serum (10%) under standard culture conditions. In some experiments HaCat cells (kindly provided by Professor Norbert Fusenig, German Cancer Research Center, Heidelberg, Germany)^63^ were used. These cells were cultured as described above. Cell lines were validated by STR DNA finger printing by the MD Anderson Cancer Center Characterized Cell Line Core using the AmpFℓSTR Identifier kit according to manufacturer instructions (Applied Biosystems cat 4322288). The STR profiles were compared to known ATCC fingerprints (ATCC.org), to the Cell Line Integrated Molecular Authentication database (CLIMA) version 0.1.200808 (http://bioinformatics.istge.it/clima/)^64^ and to the MD Anderson fingerprint database. The STR profiles matched known DNA fingerprints or were unique.

### Isolation of normal mast cells

Normal mast cells were isolated from an adult blood buffy coat obtained from an undisclosed healthy donor from the Gulf Coast Regional Blood Center (Human Research Protocol LAB-03-0390- MDACC), by partially depleting T and B cells followed by cell sorting using anti-CD34 antibody. CD34+ cells were cultured in Stempro medium enriched with IL-6, IL-3, human recombinant stem cell factor, interleukin-6, and interleukin-3. After several weeks in culture, all the viable cells stained positive for toluidine blue, tryptase, and were CD117+ (cKit).

### Cell proliferation

The rate of proliferation was analyzed using Alamar blue according to the manufacturer's instructions (Life Technologies, Carlsbad, CA, USA), as described previously.^65^ Briefly, we cultured 1 × 10^4^ and 1 × 10^3^ HMC-1 cells in 96-well plates with and without cPAF (Cayman Laboratories, Ann Arbor, MI, USA) for 24 h. After incubation, we resuspended the cells in fresh medium for another 64 h, and the rate of proliferation was analyzed every 4 h by Alamar blue. The conversion of the nonfluorescent indicator dye to a bright red color by metabolically active cells was monitored using a microplate reader. In addition, we also used the thymidine analog EdU after cells were treated with cPAF; the cells were incubated for 24 and 48 h at different concentrations of cPAF (1.25, 2.5, and 5 *μ*g/ml), followed by 2 h incubation with EdU (10 *μ*M) in cPAF-free medium. EdU-positive cells were identified using Alexa Fluor 488 Click-iT reaction kit (Life Technologies), followed by propidium iodide staining. Cells were plotted according to their DNA content and EdU-Alexa 488 staining to determine the number of cells in different phases of the cell cycle.

### Immunoblotting

Protein samples were obtained from treated and nontreated cells using RIPA lysing buffer. Briefly, cell pellets were resuspended in lysing buffer and immediately frozen at −80 ^o^C. BCA protein assay was used to determine protein concentrations (Pierce BCA Protein Assay kit, Thermo Scientific, Waltham, MA, USA). The antibodies to detect cyclin-B1, cleaved caspase 3, c-Myc, Brit1, ATR, *γ*-H2AX, p-cdc2, cyclin-D1, p-ATM (S1981), p-ATR (S428), p-ERK, ERK, GADD45, and p-p53 (S15, S9, S37, S6, S46, S20, S392, and T81) were used at 1 : 1000 dilution (Cell Signaling Labs, Danvers, MA, USA); antibodies to p21, cyclin A and CDK1 were used at 1 : 1000 dilution (Becton Dickinson, San Jose, CA, USA); antibodies to p53 (DO-1), CDK2, and CDK4 were used at 1 : 500 dilution (Santa Cruz Biotechnology, Dallas, TX, USA); the antibody to p84 was used at 1 : 1000 dilution (Genetex, Kennesaw, GA, USA); the antibody to PCNA was used at 1 : 1000 dilution (Dako Laboratories, Carpinteria, CA, USA). Natural PAF was purchased from Cayman Laboratories. Densitometry analysis, when necessary, was performed using ImageJ software (http://imagej.nih.gov/ij/).

### Analysis of the cell cycle

After each treatment cells were further incubated for 2 h with 10 *μ*M EdU in cPAF-free medium. The cells were fixed and the incorporated EdU analog was detected following the manufacturer's instructions (Click it, Invitrogen); as a final step, the cells were incubated with P.I. staining solution containing RNAse. The cells were analyzed using LSRII or Fortessa flow cytometers. Data acquisition was carried out using Diva software (BD Biosciences, San Jose, CA, USA) and further analyzed using FlowJo software (Ashland, OR, USA).

### TUNEL assay

TUNEL (Promega, Madison, WI, USA) was performed on HMC-1 cells. Briefly, DNA strand breaks were labeled with fluorescein-12-dUTP. The green fluorescence of apoptotic cells was detected by fluorescence activation cell sorting.

### Assessment of DNA damage response

HMC-1 cells were pre-cultured for 24 h with 5 *μ*g/ml of cPAF followed by UVB (200 J/m^2^; 290–320 nm) or IR (10 Gy). Cells were allowed to recover for 1 h and then were fixed with 4% paraformaldehyde for 10 min. Cells exposed to UV light were stained against p-ATR and those exposed to IR stained against p-ATM, as a measure of DNA damage response.

## Abbreviations

ATM: Ataxia telangiectasia mutated
ATR: Ataxia telangiectasia and rad related
cPAF: carbamyl platelet-activating factor
CDK: Cyclin-dependent kinase
CXCR4: Chemokine C-X-C motif receptor 4
ERK: Extracellular signal-regulated kinase
FACS: Fluorescence activated cell sorting
GADD45: Growth arrest and DNA damage-inducible 45
HMC-1: Human mast cell line 1
PAF: Platelet-activating factor
PCNA: Proliferating cell nuclear antigen
UV: Ultraviolet
