# Supplementary material for: Platelet-activating factor induces cell cycle arrest and disrupts the DNA damage response in mast cells
Source: Cell Death Dis. 2015 May 7;6(5):e1745–. doi: 10.1038/cddis.2015.115 (PMC4669695; doi:10.1038/cddis.2015.115)
Supplement: Supplementary Information [file cddis2015115x1.pdf]

Supplemental Figure 1.

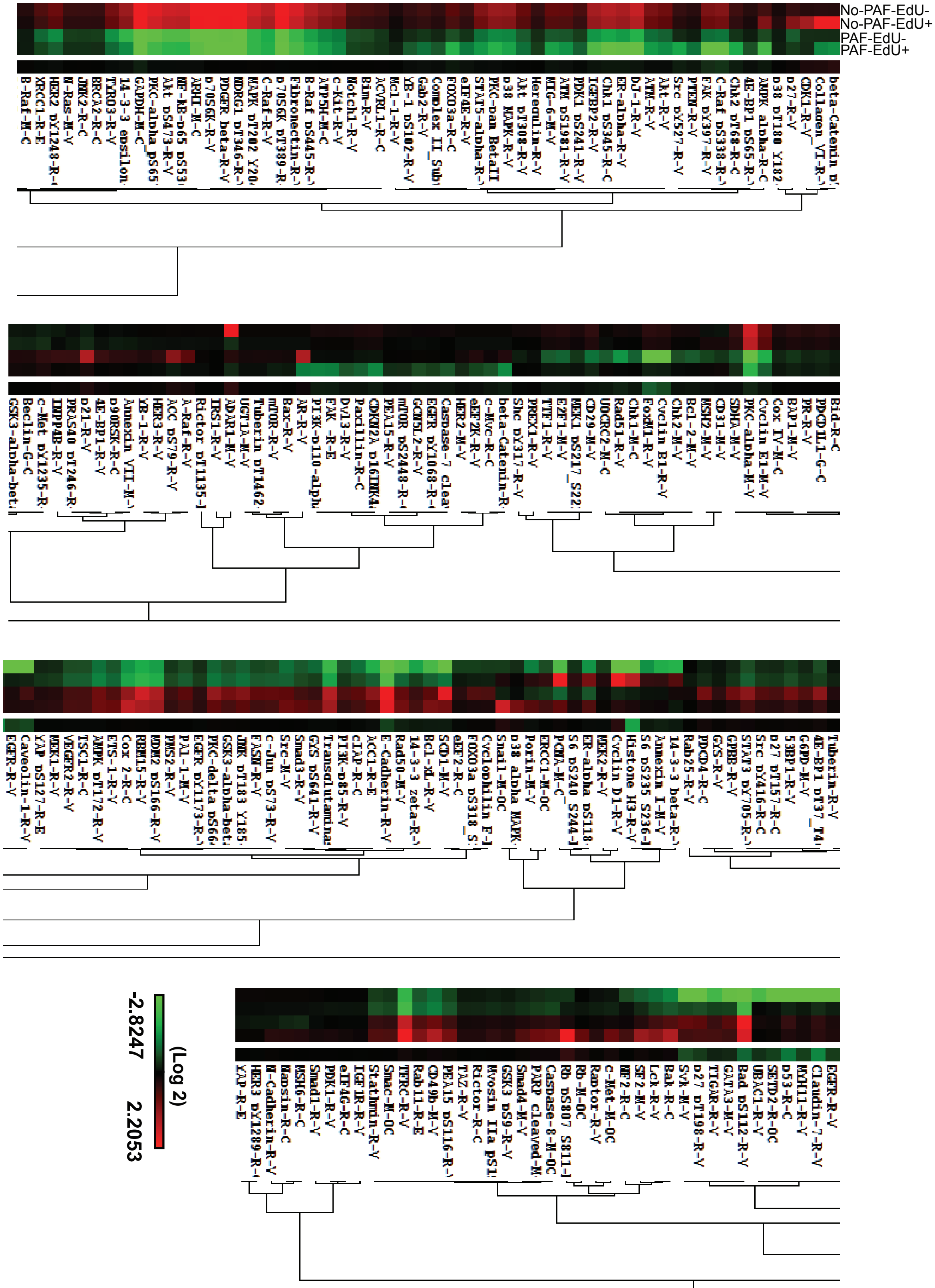

Supplemental Figure 1. Reverse phase protein assay reveals that PAF affects important components of the cell cycle. HMC-1 cells were exposed to 5 µg/ml cPAF for 24h followed by the EdU staining. We isolated EdU+ and EdU- cells by cells sorting, and protein lysates were analyzed by RPPA. PAF decrease the levels of cell cycle components cyclin B1, CDK1, cyclin D, and CDK2/4, among others.

Supplemental Figure 2

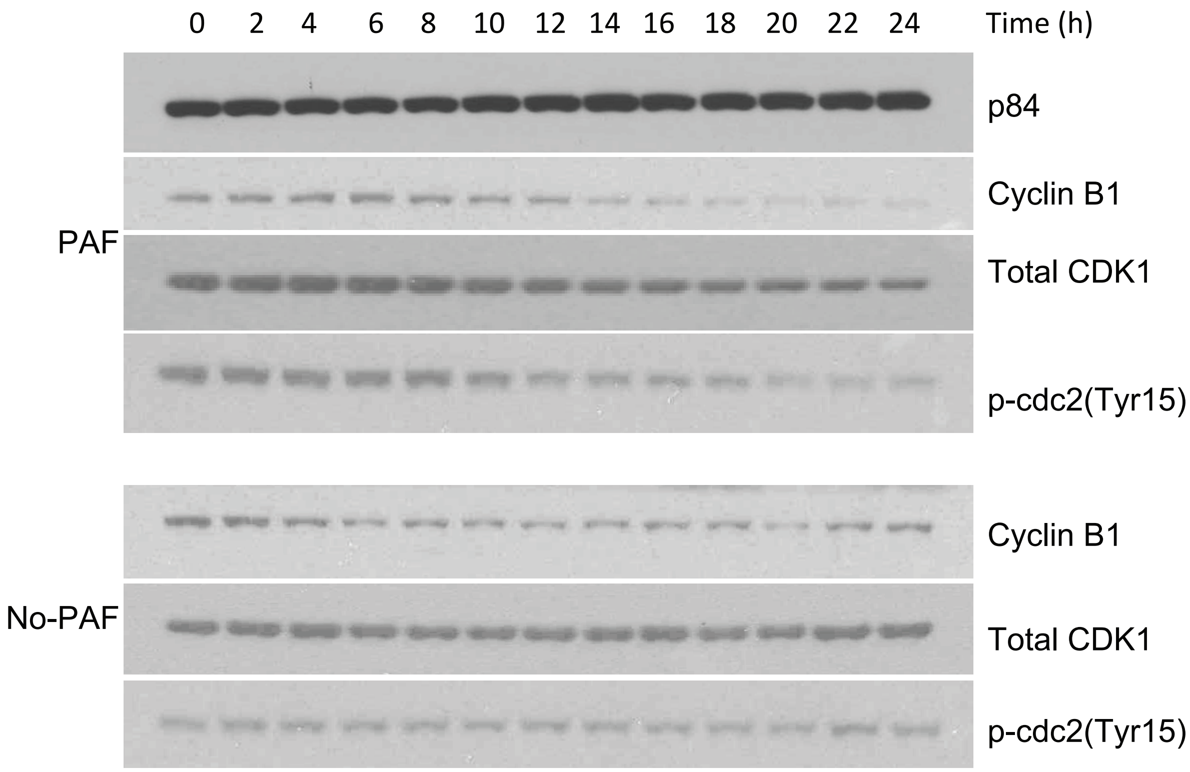

Supplemental Figure 2. Effect of PAF on the cyclin B1/CDK1 complex in synchronized HMC-1 cells by serum starvation. Cells were serum starved for 12h and then released using normal RPM1 culture medium plus cPAF. PAF-treated cells show a steady decline of cyclin B1, total CDK1, and p-cdc2(Tyr15) similar to that observed in non-synchronized cells. No significant changes in protein expression was as observed in the serum-starved vs. the non-starved samples.

Supplemental Figure 3

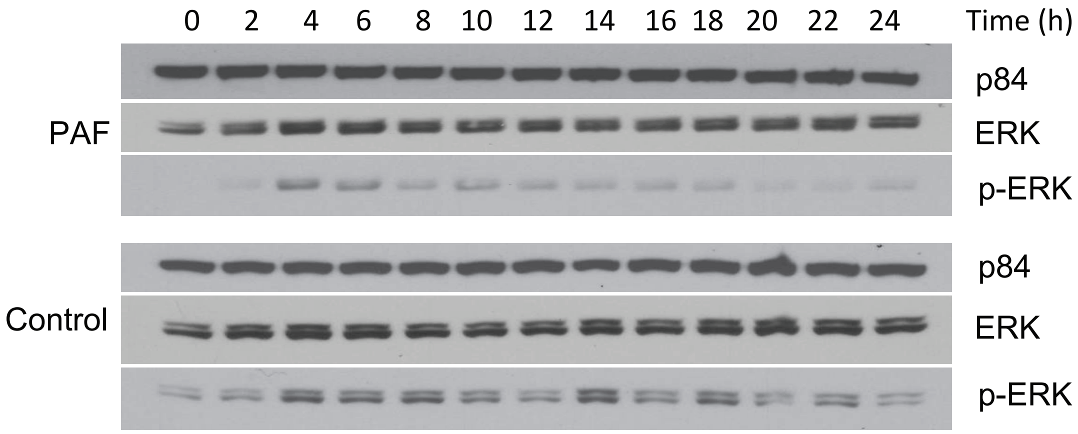

Supplemental Figure 3. PAF affects phosphorylation on ERK. Cells were collected every two hours after the addition of PAF (0 to 24h). In contrast with the control samples, a continuous reduction of p-ERK was observed starting at 6h after the addition of PAF; minimal expression was noted 20-24h post PAF-treatment.
